# Tumor microenvironment-preserving gliosarcoma organoids as an in vitro preclinical platform: a comparative analysis with glioblastoma models

**DOI:** 10.1186/s12967-025-06952-y

**Published:** 2025-08-14

**Authors:** Junseong Park, Dokyeong Kim, Hyeon-Chun Park, Minyoung Park, Songzi Zhang, Okcho Na, Youn Soo Lee, Minho Lee, Stephen Ahn, Yeun-Jun Chung

**Affiliations:** 1 Cancer Evolution Research Center, College of Medicine, The Catholic University of Korea, 222 Banpo-daero, Seocho-gu, Seoul, 06591 Republic of Korea; 2 Precision Medicine Research Center, College of Medicine, The Catholic University of Korea, Seoul, Republic of Korea; 3 Department of Microbiology, College of Medicine, The Catholic University of Korea, 222 Banpo-daero, Seocho-gu, Seoul, 06591 Republic of Korea; 4 Department of Medical Sciences, Graduate School of The Catholic University of Korea, Seoul, Republic of Korea; 5 Department of Hospital Pathology, Seoul St. Mary’s Hospital, College of Medicine, The Catholic University of Korea, Seoul, Republic of Korea; 6 Department of Life Science, Dongguk University-Seoul, Goyang, Republic of Korea; 7 Department of Neurosurgery, Seoul St. Mary’s Hospital, College of Medicine, The Catholic University of Korea, Seoul, Republic of Korea

**Keywords:** Gliosarcoma, Patient-derived organoid, Precision oncology, Single-cell RNA sequencing, Tumor microenvironment

## Abstract

**Background:**

Gliosarcoma (GS) is a rare variant of glioblastoma (GBM), characterized by biphasic glial and sarcomatous histology and poor prognosis. Despite its distinct clinical features, GS remains underrepresented in glioma research due to the lack of biologically faithful and experimentally tractable models.

**Methods:**

We established patient-derived gliosarcoma organoids (GSOs) from freshly resected tumors using a suspension culture system without enzymatic dissociation. Histological, molecular, and functional properties were evaluated using H&E staining, immunohistochemistry, whole-exome sequencing, 3D invasion assays, and single-cell RNA sequencing (scRNA-seq). Drug response assays were performed using temozolomide and ANA-12 (*NTRK2* inhibitor).

**Results:**

GSOs preserved key histological features, genetic alterations, and tumor microenvironmental cell populations from the original tumors. They stable growth and viability over extended culture periods and maintained integrity following cryopreservation and recovery, supporting their long-term utility. 3D invasion assays further revealed infiltrative behavior, consistent with the aggressive nature of GS. Histological and molecular analyses revealed that GSOs retained glial and mesenchymal differentiation, diverse non-malignant stromal cells, and case-specific somatic alterations. Comparative scRNA-seq revealed distinct transcriptional programs: GSOs were enriched for fibroblast-like and oligodendrocyte progenitor-like states, while glioblastoma organoids (GBOs) displayed astrocyte-like differentiation and high connectivity signatures. Functional assays confirmed consistent sensitivity of GSOs to temozolomide, and selective therapeutic response to *NTRK2* inhibition was observed in the GSO harboring an *NTRK2* alteration, supporting the utility for genomic-context-guided therapy.

**Conclusion:**

Together, GSOs constitute a tractable and translationally relevant model that faithfully reflects the cellular complexity, genetic landscape, and tumor-specific heterogeneity. This model addresses a critical gap in GS biology and supports its integration into precision oncology.

**Supplementary Information:**

The online version contains supplementary material available at 10.1186/s12967-025-06952-y.

## Background

Gliosarcoma (GS) is a rare and highly aggressive variant of glioblastoma (GBM), accounting for approximately 2–5% of GBMs [1, 2]. Histologically, GS is characterized by a biphasic architecture composed of both glial and mesenchymal elements, and clinically, it exhibits poor prognosis and limited responsiveness to conventional therapies such as temozolomide (TMZ) and radiation [2–4]. Despite being recognized as a distinct entity by the World Health Organization (WHO), GS remains underrepresented in large-scale glioma studies, and its molecular underpinnings are poorly understood [4]. This scarcity of knowledge, combined with the rarity and heterogeneity of disease, has hindered the development of effective, targeted treatments for GS patients.

Current preclinical models for malignant gliomas primarily rely on cell lines or patient-derived xenografts (PDXs) established from conventional GBM [5, 6]. However, these models often fail to capture the unique pathological and transcriptional features of GS, particularly its mesenchymal phenotype and sarcomatous transformation. Furthermore, PDX models are associated with considerable challenges, including high costs, time-intensive processes, and a high failure rate [7]. The absence of reliable GS-specific models severely limits mechanistic investigations and preclinical therapeutic screening. As a result, treatment strategies for GS are often extrapolated from GBM without validation in GS-relevant systems, contributing to suboptimal clinical outcomes [2, 3].

Recent advances in patient-derived organoid technology have provided a promising platform for recapitulating tumor-specific features ex vivo [8]. Organoids have been shown to preserve the histological architecture, genetic landscape, inherent heterogeneity, and drug responsiveness of various cancers, including brain tumors [9–11]. In the context of glioma research, organoid models have been successfully applied to study GBM [10], but to date, no gliosarcoma organoid (GSO) model has been systematically established or functionally validated. Moreover, traditional single-cell-based organoid models [12, 13] are unable to retain native tissue architecture, cellular complexity, and tumor microenvironmental components.

In this study, we report the first successful establishment of a tumor microenvironment-preserving organoid model from patients with GS. Our GSO model faithfully retains the histopathological and molecular features of parental tumors, including biphasic differentiation, tumor microenvironment, and patient-specific genomic alterations. Single-cell RNA sequencing (scRNA-seq) revealed distinct cellular states in GSOs relative to glioblastoma organoids (GBOs), particularly an enrichment of mesenchymal and fibroblast-like tumor cell populations. Furthermore, GSOs exhibited reproducible responses to TMZ and genotype-specific sensitivity to targeted *NTRK2* inhibition. Our findings suggest GSOs as a robust and translationally relevant in vitro platform for modeling GS biology and testing individualized therapeutic strategies.

## Methods

### Generation of tumor organoids from patient tissue

All tumor samples were pathologically diagnosed by a neuropathologist according to the 2021 WHO classification of tumors of the central nervous system. Fresh surgically resected GS and GBM tissues (Table 1) were obtained immediately after procedures at the Neuro-oncology Center of Seoul St. Mary’s Hospital and placed in sterile phosphate buffered saline. The organoid culture protocol was adapted from our previously established meningioma organoid platform, with minor modifications to optimize for GS tissue integrity and growth kinetics [9, 10]. Briefly, the resected tissue was kept at 4 ℃ and placed in Hibernate A (BrainBits LLC, Springfield, IL) supplemented with 1 × GlutaMax, 1 × PenStrep, and 1 × Amphotericin B (ThermoFisher Scientific, Waltham, MA) during tissue processing. Tumor tissue was mechanically minced into approximately 1 mm^3^-sized pieces without enzymatic digestion to preserve tissue integrity and cellular composition, followed by culture in growth medium under continuous agitation on an orbital shaker rotating at 120 rpm in a 37 ℃ and 5% CO_2_ incubator throughout the maintenance. Growth medium is a mixture of 50% DMEM-F12 and 50% Neurobasal supplemented with 1 × GlutaMax, 1 × NEAAs, 1 × PenStrep, 1 × N_2_ supplement, 1 × B27 w/o vitamin A supplement, 1 × 2-mercaptoethnaol, and 2.5 μg/ml human insulin; human insulin solution was purchased from Sigma-Aldrich (St. Louis, MO) and all others were obtained from Themo Fisher Scientific. For cryopreservation of organoids, the freezing medium consisted of the growth medium with 10 μM Y-27632 (Tocris Bioscience, Bristol, UK) and 10% DMSO (Sigma-Aldrich). For recovery, cryovials were rapidly thawed, and the organoids were resuspended in growth medium supplemented with 10 μM Y-27632 and 1% Matrigel (Corning Inc., Corning, NY). After two weeks, organoids were stabilized in growth medium without Y-27632 and 1% Matrigel and then subjected to downstream assays. The study was conducted according to the guidelines of the Declaration of Helsinki, and approved by the Institutional Review Board of Seoul St. Mary’s Hospital (approval number: KC21TISI0793). Tumor samples were collected following the acquisition of informed consent from patients.

**Table 1:** Clinical characteristics of patients included in this study

Sample	Age	Sex	Diagnosis	Grade	Tumor location	*IDH1*	1p19q	*TERT*	*MGMT*
GSO21-01	86	F	Gliosarcoma	IV	Left temporal	Wild-type	Intact	Mutant	Unmethylated
GSO22-01	55	F	Gliosarcoma	IV	Right temporal	Wild-type	Intact	Mutant	Methylated
GBO21-07	66	M	Glioblastoma	IV	Left occipital	Wild-type	Intact	Mutant	Unmethylated
GBO22-13	84	M	Glioblastoma	IV	Right temporal	Wild-type	Intact	Mutant	Methylated
GBO23-01	15	M	Glioblastoma	IV	Left frontal	Wild-type	Intact	Wild-type	Unmethylated
GBO23-02	58	F	Glioblastoma	IV	Right fronto-temporal	Wild-type	Intact	Mutant	Unmethylated
GBO24-01	81	F	Glioblastoma	IV	Left frontal	Wild-type	Intact	Mutant	Unmethylated

### Measurement of organoid size and viability

Single organoids were transferred into individual wells of a 96-well culture plate. The growth of organoids was assessed using WST assays with Cell Counting Kit-8 (CCK-8) reagents (Dojindo Laboratories, Kumamoto, Japan). The plates were incubated with 10% CCK-8 reagent for 1.5 h, and absorbance was measured at 450 nm using a microplate reader (SYNERGY H1, Bio-Teck, Winooski, VT). The 2D area of each organoid was quantified using ToupView software by carefully outlining the organoid boundaries. Images of individual organoids were captured weekly using a bright-field microscope.

### Histology and immunostaining

Organoids were fixed in 4% paraformaldehyde within 1 h, placed in a plastic cryomold, and snap frozen in tissue freezing medium on dry ice. The sections (8–10 μm thick) of GSOs were sliced using a cryostat (Leica, Wetzlar, Germany). Hematoxylin–eosin (H&E) staining, immunofluorescence (IF), and immunohistochemistry (IHC) were performed following a widely used protocol described elsewhere. Special stains, including Masson’s trichrome and reticulin, were performed to confirm the collagenic nature. The slides were scanned by Pannoramic SCAN II (3DHISTECH Ltd, Budapest, Hungary) and representative images were captured by image viewing software (CaseViewer and Automated Slide Analysis Platform). IF staining images were captured using a Zeiss LSM 800 confocal microscope with 5 × and 40 × objective lens with Zen software (Zeiss, Jena, Germany). Cell nuclei were counter-stained with Hoechst (Invitrogen, Waltham, MA) for IF images. Details of the antibodies used for immunostaining are provided in Additional file 1: Table S1.

### 3D invasion assay

Each organoid was embedded in matrix composed of type I collagen (Nitta Gelatin, Osaka, Japan) and Matrigel. The matrix mixture was prepared from collagen type I with Matrigel (1:1) in the 2 × Ham’s F12 medium on ice. After that, the pH was adjusted by adding 10% reconstitution buffer (0.002 g/mL NaHCO_3_, 0.0047 g/mL HEPES, and 0.005 N NaOH). Matrix solution (100 μL) was directly put into a 96-well plate and the single organoids were embedded into the matrix prior to gelation. Culture medium and fetal bovine serum (1:1 ratio) were added over the gelled matrix. The relative invasion area of each organoid was quantified by normalizing with the occupied area at 0 d [14].

### Organoid cytotoxicity assay

The quantification of plasma membrane damage and cytotoxicity was assessed using a lactate dehydrogenase (LDH) assay with LDH-Glo™ cytotoxicity Assay Kit (Promega Corporation, Fitchburg, WI). Single organoids were transferred to a 48-well plate with fresh medium and incubated for 72 h with *NTRK2* antagonist (ANA-12; MedChemExpress, NJ) or TMZ (Selleckchem, Houston, TX). After the incubation period, the culture medium from each organoid was diluted 20-fold in PBS and subsequently mixed with the LDH detection reagent at 1:1 ratio. Luminescence was recorded after 1 h incubation at room temperature (RT). Following this, 0.2% triton X-100 was added for 30 min to induce 100% cell death of organoids, and the cytotoxicity of each organoid was normalized by these positive controls.

Live/dead cells were stained with acridine orange (AO)/propidium iodide (PI) using Cyto3D Live/Dead assay Kit (TheWell Bioscience Inc., Monmouth Junction, NJ). PI penetrates only the membranes of dead cells, producing red fluorescence. AO is permeable to both live and dead cells, but its signal is quenched in non-viable cells due to fluorescence resonance energy transfer when co-localized with PI, eventually emitting green fluorescence in only viable cells. Each organoid was transferred into 96-well plates and incubated with 2% Cyto3D reagent for 10–15 min in 5% CO_2_ and at 37 ℃. The cell viability was observed using a fluorescence microscope.

### Whole-exome sequencing (WES)

DNA was extracted from approximately ten GSOs (passages > 3) and their parental tumor and normal tissues, comprising two patient-derived pairs and six samples in total. WES was performed using the Illumina NovaSeq platform (Illumina, San Diego, CA) with Agilent SureSelect XT human all exon kit (Agilent Technologies Inc., Santa Clara, CA). The Burrows-Wheeler Aligner (v0.7.17) [15] was used to align raw sequencing data onto the GRCh38 human reference genome. Preprocessing of aligned data was performed under the somatic short variant discovery (single nucleotide variants, insertion, and deletion) in Best Practices Workflows of the genome analysis toolkit (GATK) version 4.4.0.0 [16, 17]. Somatic variants were identified using GATK’s Mutect2 algorithm by comparing tumor tissues or GSOs to their matched normal tissues (Additional file 1: Table S2). Variant annotation was subsequently performed using ANNOVAR [18] with standard reference databases. Variant filtering was applied using the following criteria: sequencing depth below 20 reads, variant allele frequency (VAF) less than 5%, fewer than five alternative reads, variants in non-exonic regions, variants with unknown exonic functions, and variants with allele frequencies greater than 1% in East Asian population databases were excluded. After applying these filters, 320 variants that passed Mutect2 quality criteria were retained. Variants not flagged as “PASS” by Mutect2 but consistently identified in paired tumor and organoid samples were rescued, resulting in a final set of 650 somatic variants (Additional file 1: Fig. S2A). Visualization of somatic variants was conducted using maftools [19] in R to provide comprehensive graphical summaries of variant distributions. Copy number alterations (CNAs) were analyzed using FACETS (0.6.1) [20] with paired tumor-normal WES data as input.

### Dissociation of organoids and scRNA-seq

Ten GBOs per experimental group were minced into fragments smaller than 1 mm^3^ using a sterile razor blade. The tissue fragments were enzymatically dissociated in a shaking incubator at 37 ℃ and 120 rpm for 30 min. The dissociation medium comprised a 1:1 mixture of DMEM/F12 and Neurobasal medium, supplemented with Liberase TM (1.25 mg/mL; Roche, Basel, Switzerland) and DNase I (5000 U/mL; Roche). Following digestion, the cell suspensions were filtered through a pre-washed 40-μm cell strainer and washed with 15 mL of the same 1:1 MEM/F12 and Neurobasal medium mixture. Cells were then pelleted by centrifugation at 300 g for 10 min at 4 ℃ and subsequently resuspended in fresh medium. Cell count and viability were assessed using the Countess Automated Cell Counter (ThermoFisher Scientific), with all samples exhibiting viabilities greater than 70%. The dissociated cells were washed, resuspended at a concentration of ~ 1000 cells/μL, and used for generating scRNA-seq libraries with Chromium Single-cell 3’ Reagent Kits version 3 (10 × Genomics, Pleasanton, CA) following the manufacturer’s instructions. Sequencing data were aligned to the human reference genome (GRCh38) and processed using the Cell Ranger 7.2.0 pipeline (10 × Genomics). After doublet removal using DoubletFinder [21], low-quality cells with fewer than 1500 unique molecular identifiers (UMIs) or greater than 20% mitochondrial genes were excluded (Additional file 1: Fig. S3). All individual datasets were then integrated with batch correction using the RPCA algorithm. Subsequent analyses, including normalization, UMAP dimensionality reduction, and differentially expressed gene (DEG) analyses, were conducted with the R (v4.4.2) package Seurat (v5.2.1) [22] to identify, characterize, and visualize clusters as previously described [23–25]. The clusters were manually annotated by comparing the canonical marker genes, DEGs, and cellular states [26–28] for each cluster. The inferCNV tool (v1.22.0) [29] was used to generate inferred single-cell CNA profiles, and tumor cells were determined mainly by chromosome 7 gain (Additional file 1: Fig. S4A). Enrichment analyses were performed using the R package UCell [30], using gene sets derived from the c2 canonical pathways of MSigDB [31].

### Statistical analysis and software

Student’s *t* test, one-way ANOVA, and repeated measured ANOVA were carried out using GraphPad Prism (v9.5.1) to calculate significance. Results are expressed as mean ± standard error of the mean (SEM).

## Results

### Conservation of histological and genomic features in GSOs

**Fig. 1:**
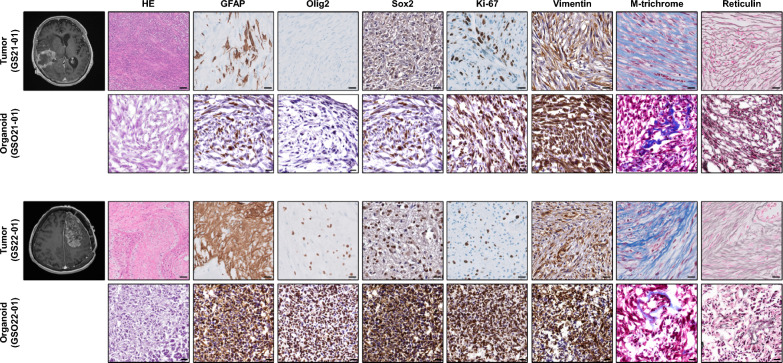
Radiographic and histological images of GSOs and their parental tumors. Magnetic resonance images, H&E staining, and IHC using antibodies against GFAP, Olig2, Sox2, Ki-67, and vimentin, and special stain images using Masson’s trichrome and reticulin in GSOs and their parental tumors. Scale bars represent 20 µm (magnification × 40)

To establish a biologically faithful in vitro model of GS, we generated two patient-derived GSOs using freshly resected tumor tissue (Table 1 and Fig. 1). Upon organoid formation, tissue morphology was maintained with remarkable fidelity. GSOs recapitulated the biphasic histology characterized by intermixed glial (GFAP) and sarcomatous (Mason’s trichrome, reticulin, and vimentin) components (Fig. 1 and Additional file 1: Fig. S1). Notably, GSO21-01 exhibited no detectable Olig2 expression and low Sox2 levels, mirroring the features of GS21-01, whereas GS/GSO22-01 displayed the opposite pattern. Ki-67 staining revealed high proliferative activity in both tumors and organoids, supporting the proliferative feature of the model.

**Fig. 2:**
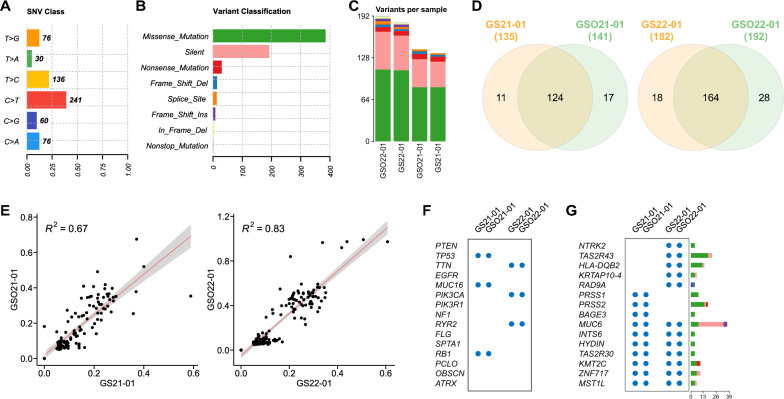
WES of GSOs and corresponding parental tumors. **A**, **B** The number of each class of single nucleotide variants **A** and variant types **B** for all GS and GSO samples. **C** The number of variants, colored by variant classification in **B**, for each sample. **D** Venn diagrams showing the number of shared and unique variants between corresponding samples. **E** Scatter plots representing VAFs of shared mutations between corresponding samples. Pearson’s correlation coefficients were displayed as *R*^2^. **F** Variants were mapped to frequently mutated genes in GBM based on cBioPortal TCGA datasets. **G** Representative genes harboring somatic variants. Variant types defined in **B** are displayed

Next, we performed WES on paired parental tumors and GSOs to assess whether GSOs retained the genomic landscapes of their corresponding parental tumors (Additional file 2: Data S1–S4 and Fig. 2A, B). While the two patient-derived samples exhibited distinct genomic landscapes (Additional file 1: Fig. S2B), most variants were shared between GSOs and their corresponding tissues (Fig. 2C, D), and quantitative analysis of VAFs revealed a strong correlation between them (Fig. 2E), indicating preservation of the clonal architecture. Additionally, CNAs detected in the parental tumors were also identified in the corresponding GSOs (Additional file 1: Fig. S2C, D), indicating the consistency of GSOs in recapitulating tumor-specific CNA profiles. Notably, genetic alterations encompassed both mutations frequently reported in GBM (Fig. 2F) and case-specific mutations (Fig. 2G). All these histological and genetic concordances between patient tumors and derived organoids across samples underscores the robustness of our GSO model.

### Functional characterization of GSOs in vitro

**Fig. 3:**
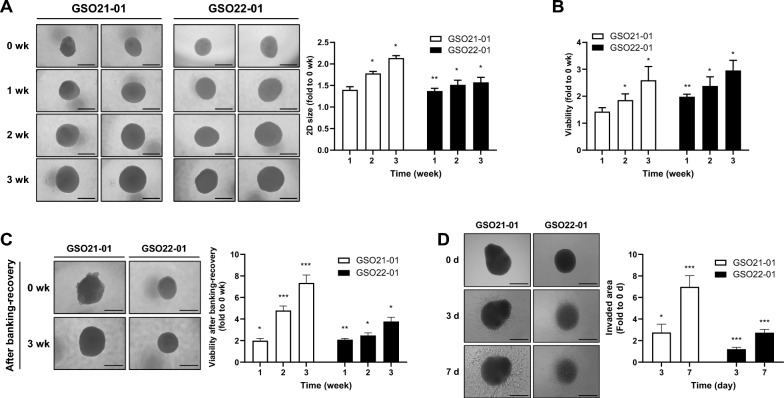
Functional characteristics of GSOs. **A** Representative bright-field microscopy images of individual growing GSOs over a 3-weeks culture period. The two-dimensional size of the GSOs were quantified using ToupView software. **B** GSO viability was assessed weekly over 3 weeks using the WST assays (n = 8, GSO21-01; n = 5, GSO22-01). **C** Morphology and viability of GSOs post-banking and recovery were evaluated via bright-field imaging and the WST assays (n = 6, GSO21-01; n = 4, GSO22-01). **D** Invasiveness of individual GSOs (n = 8) was measured at 3 d and 7 d using 3D invasion assays, and representative images were captured by bright-field microscopy. The invaded areas were quantified using ToupView software. For **A**–**D**, all bright-field images include scale bars representing 500 µm, and repeated measured ANOVA was performed to evaluate statistical significance compared with 0 wk or 0 d control (*P < 0.05, **P < 0.01, ***P < 0.001)

To evaluate the in vitro functionality of GSOs, we monitored their growth and viability over a three-week culture period. Organoid size was measured longitudinally, revealing a progressive increase (Fig. 3A). Consistently, viability assays demonstrated that GSOs maintained high viability and proliferative capacity throughout the culture duration (Fig. 3B). Notably, both GSOs successfully re-established 3D structures with preserved viability and morphology after cryopreservation and subsequent recovery (Fig. 3C), suggesting the robustness of the GSO biobanking process. Furthermore, we evaluated the invasive potential of GSOs using a 3D invasion assay. GSOs exhibited active invasion into the surrounding matrix, characterized by radial extension of cellular protrusions (Fig. 3D). Collectively, these results highlight the proliferative, viable, and invasive properties of GSOs in vitro, supporting their utility as a functional platform for GS research.

### Comparative analysis of GSOs and GBOs

**Fig. 4:**
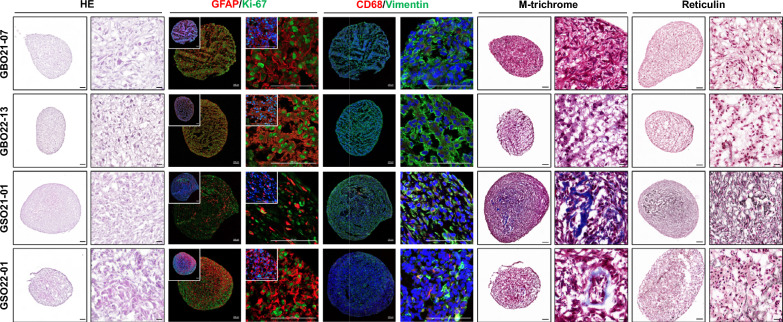
Comparative histological analysis of GSOs and GBOs. Two GBOs (21-07 and 22-13) and two GSOs (21-01 and 22-01) were analyzed by H&E staining, IF using antibodies against GFAP, Ki-67, CD68, and vimentin, and special staining with Masson’s trichrome and reticulin. Cell nuclei were counter-stained with Hoechst (blue). Scale bars for H&E, Masson’s trichrome, and reticulin images represent 100 µm (left panels) and 20 µm (right panels). Scale bars for IF images represent 100 µm

To compare the distinct features of GSOs relative to GBOs, we additionally established GBO lines from GBM patient tissues using the same culture methods employed for GSOs (Additional file 1: Fig. S3). Although GBOs also exhibited similar functional properties, including high viability, proliferative capacity, and invasive potential, IHC and histological analyses revealed notable differences between GSOs and GBOs. While both models demonstrated GFAP positivity indicative of glial lineage, GSOs consistently exhibited stronger mesenchymal signatures, as evidenced by extensive deposition of extracellular matrix (ECM) components highlighted by Mason’s trichrome and reticulin staining. Additionally, CD68 staining revealed pronounced infiltration of microglia/macrophages within GSOs (Fig. 4).

**Fig. 5:**
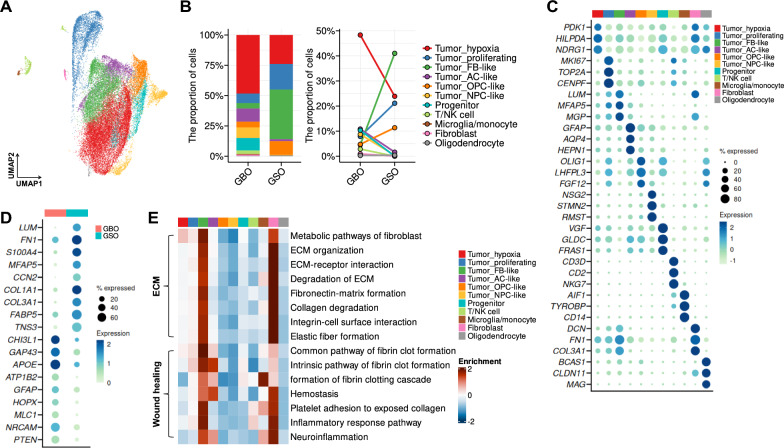
scRNA-seq analysis of GSOs and GBOs. **A** UMAP plot colored by clusters. **B** Bar plot and line graph showing the proportion of clusters across groups. **C**, **D** Dot plot showing the average expression of representative genes across clusters **C** and groups **D**. **E** Heat map obtained from the enrichment analysis for gene sets associated with canonical pathways

To further examine the cellular heterogeneity and transcriptional programs underlying the histological variations described above, we analyzed single-cell gene expression profiles of four GBO and two GSO lines, comprising a total of 47,863 cells (Additional file 1: Fig. S4). Both GSOs and GBOs encompassed not only tumor cells but also components of the tumor microenvironment, including microglia/monocyte and oligodendrocyte (Fig. 5A and Additional file 1: Fig. S5A, B). Among these 11 distinct cell annotations, GSOs exhibited a markedly higher proportion of fibroblast-like tumor cells (Tumor_FB-like) compared to GBOs (Fig. 5B), characterized by the expression of fibroblast and cancer-associated fibroblast (CAF) marker genes (Fig. 5C, D and Additional file 1: Fig. S5C). In addition, GSOs demonstrated higher oligodendrocyte progenitor cell (OPC)-like state marker genes [26], whereas GBOs showed strong connectivity signatures [27] and astrocyte (AC)-like states (Fig. 5D). Notably, “Tumor_FB-like” cluster exhibited strong enrichment for ECM organization- and wound healing-associated gene sets, similar to the “Fibroblast” cluster (Fig. 5E). Together, these histological and transcriptomic comparisons delineate a clear distinction between GSO and GBO models, underscoring the mesenchymal dominance of GSOs and the need for tailored therapeutic strategies for GS patients.

### Evaluation of therapeutic strategies in GSOs

**Fig. 6:**
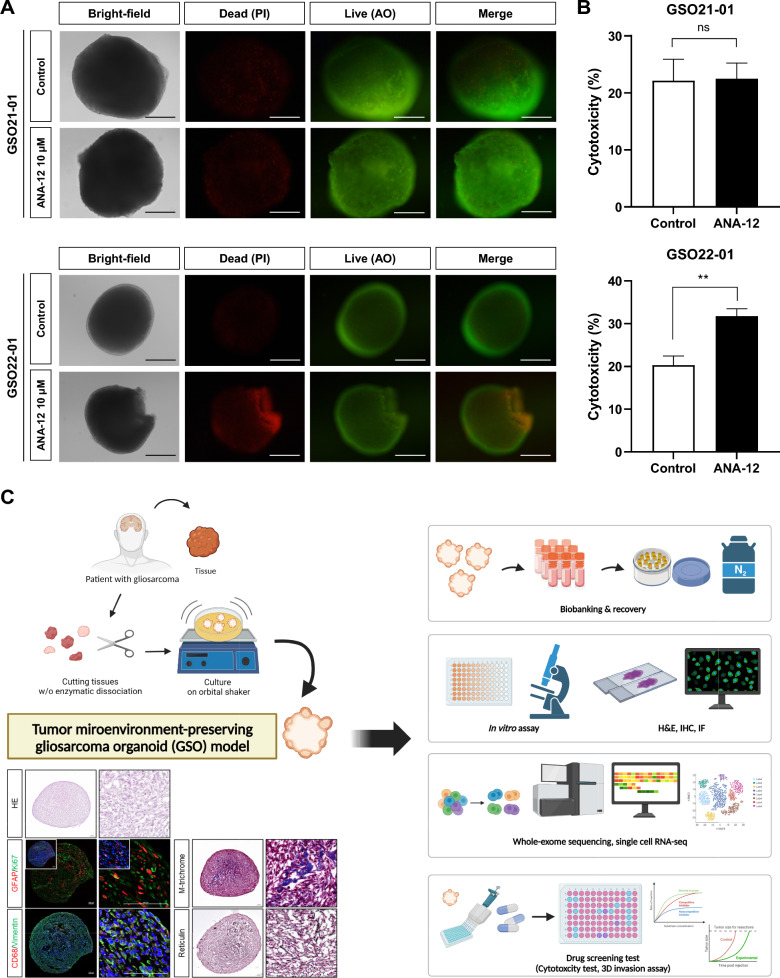
Therapeutic response to *NTRK2* inhibition in GSOs. Both GSO lines were treated with 10 µM ANA-12 for 72 h. **A** Live/dead fluorescence imaging. Dead cells were labeled with PI (red), while viable cells were labeled with AO (green). **B** LDH assays were conducted to measure cytotoxicity of ANA-12 in GSOs (n = 6). Triton X-100 was used as a positive control. Statistical significance was determined using student *t*-test (**P < 0.01). **C** Schematic summary of the study. A patient-derived GSO model was successfully established, incorporating tumor cells and diverse microenvironmental components, including collagen and reticular fibers. The model was amenable to cryopreservation and recovery, and showed histological and transcriptional features that distinguish GSOs from GBM organoids. The GSO platform was further applied to WES-based precision oncology

To evaluate the utility of GSOs as a preclinical model, we performed drug sensitivity assays using agents with clinical relevance to glioma. TMZ, the current standard-of-care chemotherapy for GBM, elicited a consistent therapeutic response across both GSO lines, as evidenced by reduced viability and invasiveness, along with changes in marker expression following treatment (Additional file 1: Fig. S6). Next, we assessed the efficacy of ANA-12, a selective *NTRK2* inhibitor, based on WES data (Fig. 2G). Notably, only the GSOs harboring an *NTRK2* mutation (GSO22-01) responded to ANA-12 treatment, exhibiting significant suppression of viability and organoid integrity (Fig. 6A, B). In contrast, the *NTRK2*-wildtype GSO (GSO21-01) displayed no appreciable sensitivity (Fig. 6A, B), highlighting the predictive power of GSOs in targeted therapy. Taken together, our GSO model faithfully reflects genomic mutations present in the parental tumors, enabling the identification and application of personalized therapeutic strategies. Furthermore, GSOs hold significant potential as an in vitro preclinical platform for predicting the treatment response in GS patients (Fig. 6C).

## Discussion

In this study, we established and comprehensively characterized patient-derived GSOs. To preserve native cell–cell architecture and the tumor microenvironment, we dissected tumor tissues into small fragments without enzymatic dissociation. In addition, we employed a liquid medium-based culture system without Matrigel embedding, resulting in a protocol that is easy-to-handle, cost-efficient, and scalable. These GSOs preserved the biphasic architecture of GS, including mesenchymal and glial components, and retained key somatic mutations, CNAs, and diverse tumor microenvironmental cell types from the parental tissues. Furthermore, GSOs could maintain their integrity after long-term culture and cryopreservation-recovery cycles, supporting biobanking and reproducible future applications, a crucial feature for developing rare tumor models. In contrast to GBOs, GSOs exhibited a mesenchymal-dominant transcriptional profile, including a fibroblast-like tumor cell population and strong enrichment for ECM-associated programs. Functionally, GSOs responded to standard chemotherapy with TMZ and enabled genotype-specific therapeutic evaluation, as demonstrated by selective sensitivity to *NTRK2* inhibition in an *NTRK2*-altered organoid. Collectively, our findings position GSOs as a novel and robust platform for modeling GS biology and testing individualized therapies.

In the context of GBM organoid research, our GSO model offers notable advantages over previously reported systems. Unlike cerebral organoid-based tumor models, which rely on implantation of glioma stem cells into cerebral organoids [12], our GSO model is directly established from freshly resected patient tumors. This approach enables the preservation of both malignant and non-malignant components of the native tumor microenvironment without requiring artificial reconstruction or host-derived scaffolds. Furthermore, compared to the Matrigel-based GBM organoid models [13], which involves enzymatic dissociation of tumor tissue followed by re-embedding glioma stem-like cells into extracellular matrix to maintain growth and stemness, our suspension-based GSO system circumvents both dissociation and matrix embedding steps. This preserves the native tissue architecture, cellular heterogeneity, and microenvironmental complexity, which are often lost in conventional single-cell-based cultures. In addition, our model offers a simplified, scalable, and time-efficient platform that is well-suited for high-throughput functional assays and precision oncology applications. Although previous studies have described organoid models for GBM and meningioma [9–11], GS has remained largely unmodeled despite its distinct pathological and molecular features. Traditional GBM-derived models, including tumorspheres [14, 32, 33] and xenografts [34, 35], rarely capture the mesenchymal transition or the biphasic morphology characteristic of GS. While pharmacokinetic parameters, such as drug delivery, metabolism, and clearance, are inherently uncaptured in GSOs, as in most current organoid and 3D culture models due to the absence of vasculature, blood–brain barrier, and systemic circulation, the preservation of ECM components in GSOs may better recapitulate in vivo diffusion barriers compared to models lacking these features. Therefore, the GSO model is best positioned for therapeutic target discovery, functional validation, and drug response assessment, rather than pharmacokinetic simulation. Future studies incorporating pharmacokinetic assessments of drug diffusion gradients, intracellular uptake, and metabolic clearance would further strengthen the translational relevance of GSOs. GSOs offer advantages even over animal models, which often involve interspecies differences, require immunocompromised hosts, and are time- and resource-intensive. While in vivo models better mimic systemic physiology, GSOs offer a faster, human-relevant platform for mechanistic studies and drug screening without the redundancy of animal engraftment.

In our comparative analyses, we observed that GSOs differ substantially from GBOs not only in histological architecture but also in their single-cell transcriptomic landscape. Enrichment of fibroblast-like tumor cells in GSOs is consistent with previous reports, and they are associated with glial-like wound response [36]. Additionally, GSOs exhibited an increased proportion of OPC-like tumor states, while AC-like states were dominated in GBOs with high connectivity scores (Fig. 5B). This distinction is highly relevant in light of recent studies that have classified GBM into dynamic cellular states with lineage-like transcriptional programs [26]. Among these, OPC-like states are associated with a proliferative, progenitor-like phenotype, whereas AC-like states tend to exhibit more differentiated features and are linked to tumor regions with relatively low mitotic activity and higher intratumoral connectivity [26, 27]. Our observation that GSOs are enriched for OPC-like signatures suggests that GS may harbor a more proliferative and developmentally primitive cellular composition compared to GBM. This may partly explain the rapid growth and aggressive mesenchymal features observed in GSOs. Previous studies have shown that mesenchymal stem-like cells promote invasion and therapeutic resistance in GBM tumorspheres [35, 37], supporting the functional significance of mesenchymal and progenitor-like states in glioma progression. The differential enrichment of cellular states underscores not only the biological divergence between GS and GBM but also the potential for distinct therapeutic vulnerabilities, further highlighting the inadequacy of applying GBM-based models to study GS. Additionally, the origin and developmental trajectory of GS, particularly the emergence of mesenchymal components within a glial tumor, remains largely undefined, in contrast to the more studied subventricular zone-derived GBM models [38]. Our GSO model offers a valuable platform for exploring these questions by enabling the banking of GSOs from multiple patients with this rare tumor subtype. As large-scale, unbiased sampling in GS is nearly impractical due to its low incidence, this organoid biobanking provides a practical means to focus on GS pathogenesis and potentially uncover tumor lineage relationships or cells of origin through future integrative analyses.

A key strength of our model is its applicability to precision oncology, as demonstrated by the *NTRK2* inhibitor experiment. NTRK gene fusions and mutations have been identified across various cancers, including lung cancer, colorectal cancer, and pediatric glioma, and serve as actionable targets for small-molecule inhibitors such as larotrectinib and entrectinib [39, 40]. Furthermore, several clinical trials are currently underway evaluating NTRK inhibitors in diverse types of cancers (NCT04655404, NCT04879121, NCT05783323), underscoring the growing clinical interest in targeting NTRK. In our study, only the GSO harboring an *NTRK2* alteration responded to ANA-12, while the *NTRK2*-wild-type GSO was resistant (Fig. 6). This finding exemplifies the value of GSOs for functionally validating patient-specific genomic alterations and supports the integration of GSOs into genotype-matched preclinical testing. As standard treatment regimens for GS are currently extrapolated from GBM despite their biological distinctions, actionable mutations such as *NTRK2* can be prioritized in therapeutic strategies. We highlight the value of GSO biobanks for modeling inter-patient variability, lineage plasticity, and therapy-induced adaptation, which support translational applications such as biomarker discovery. This platform may also support patient stratification, biomarker-guided trial design, and high-throughput drug screening [41, 42], thereby advancing the integration of functional evidence into clinical decision-making for rare tumors like GS.

While promising, several limitations should be acknowledged. (1) The number of organoid lines analyzed in this study is limited due to the extreme rarity of the disease, which constrains the availability of surgical specimens. Nevertheless, our study demonstrates that GSOs could be successfully generated from all GS cases attempted (2 of 2), suggesting technical reproducibility. Furthermore, it is important to note that the success rate of GBO establishment using a similar culture protocol in our previous work and those of other groups [10] exceeds 90%, suggesting that the GSO model is likely to be reproducible when applied to additional GS cases. Although GSO21-01 exhibited more aggressive phenotypes in vitro, including higher invasiveness and proliferation, than GSO22-01 (Fig. 3), we cannot determine molecular causal factors contributing to this behavior. Expanding the cohort through multi-institutional collaboration will be essential to capture the full extent of inter-patient heterogeneity in GS. (2) Furthermore, although GSOs recapitulate many intrinsic features of the parental tumors, they do not fully reflect the complex tumor microenvironment, particularly elements such as vasculature and adaptive immune cells. Integration with perfusion-based organ-on-chip systems or vascularized 3D models could overcome these limitations supporting vascular mimicry and drug delivery dynamics. Incorporation of co-culture systems with immune or stromal cells, or integration of perfusable vascular scaffolds or xenograft models, may further enhance the physiological relevance of the GSO platform, extending its utility to immuno-oncology or anti-angiogenic drug testing contexts [43, 44]. (3) While the GSOs demonstrated phenotypic stability during early-passage culture and following cryopreservation, we acknowledge the possibility of long-term culture adaptations. Although several studies have shown that short- to mid-term culture does not result in significant genetic drift, prolonged in vitro propagation may promote selection of subclones adapted to culture conditions or lead to transcriptional and epigenetic shifts. Future studies will be needed to characterize these potential adaptations and selective pressures over extended culture periods through serial genomic, transcriptomic, and functional profiling. Longitudinal and multi-regional sampling of tumor tissues may also better capture the temporal and spatial heterogeneity of GS, including clonal diversity and evolutionary dynamics under therapeutic pressure [42]. Future efforts incorporating GSOs derived from matched pre- and post-treatment samples could enable modeling of therapy-induced adaptation and clonal evolution over time.

## Conclusion

Expanding the GSO biobank will be essential to further advance the translational potential of this model, enabling deeper exploration of tumor subtypes, therapy response, and inter-patient variability. These models may support functional genomic interrogation, high-throughput drug screening, and the study of therapy-induced adaptation or lineage plasticity. As precision oncology increasingly demands patient-specific functional evidence, GSOs provide a valuable bridge between molecular profiling and treatment decision-making. By capturing key histological, molecular, and microenvironmental features of GS, this work lays the foundation for incorporating GSOs into personalized therapeutic pipelines and for advancing targeted strategies in GS, which remains one of the most challenging tumors in neuro-oncology.

## Supplementary Information

Supplementary Material 1.

Supplementary Material 2.

## Data Availability

The WES datasets of the current study are available in the NCBI SRA database under accession number PRJNA1263112.

## Abbreviations

AC: Astrocyte
AO: Acridine orange
CAF: Cancer-associated fibroblast
CAN: Copy number alteration
DEG: Differentially expressed gene
DMSO: Dimethyl sulfoxide
ECM: Extracellular matrix
FB: Fibroblast
GATK: Genomic Analysis Tool Kit
GBM: Glioblastoma
GBO: Glioblastoma organoid
GS: Gliosarcoma
GSO: Gliosarcoma organoid
H&E: Hematoxylin & eosin
IF: Immunofluorescence
IHC: Immunohistochemistry
LDH: Lactate dehydrogenase
OPC: Oligodendrocyte progenitor cell
PDX: Patient-derived xenograft
PI: Propidium iodide
scRNA-seq: Single-cell RNA sequencing
TMZ: Temozolomide
UMI: Unique molecular identifier
VAF: Variant allele frequency
WES: Whole-exome sequencing
WHO: World Health Organization
