# Supplementary material for: Tumor microenvironment-preserving gliosarcoma organoids as an in vitro preclinical platform: a comparative analysis with glioblastoma models
Source: J Transl Med. 2025 Aug 14;23:915. doi: 10.1186/s12967-025-06952-y (PMC12355768; doi:10.1186/s12967-025-06952-y)
Supplement: Supplementary file 1 — Supplementary Material 1. [file 12967_2025_6952_MOESM1_ESM.pdf]

Supplementary Information

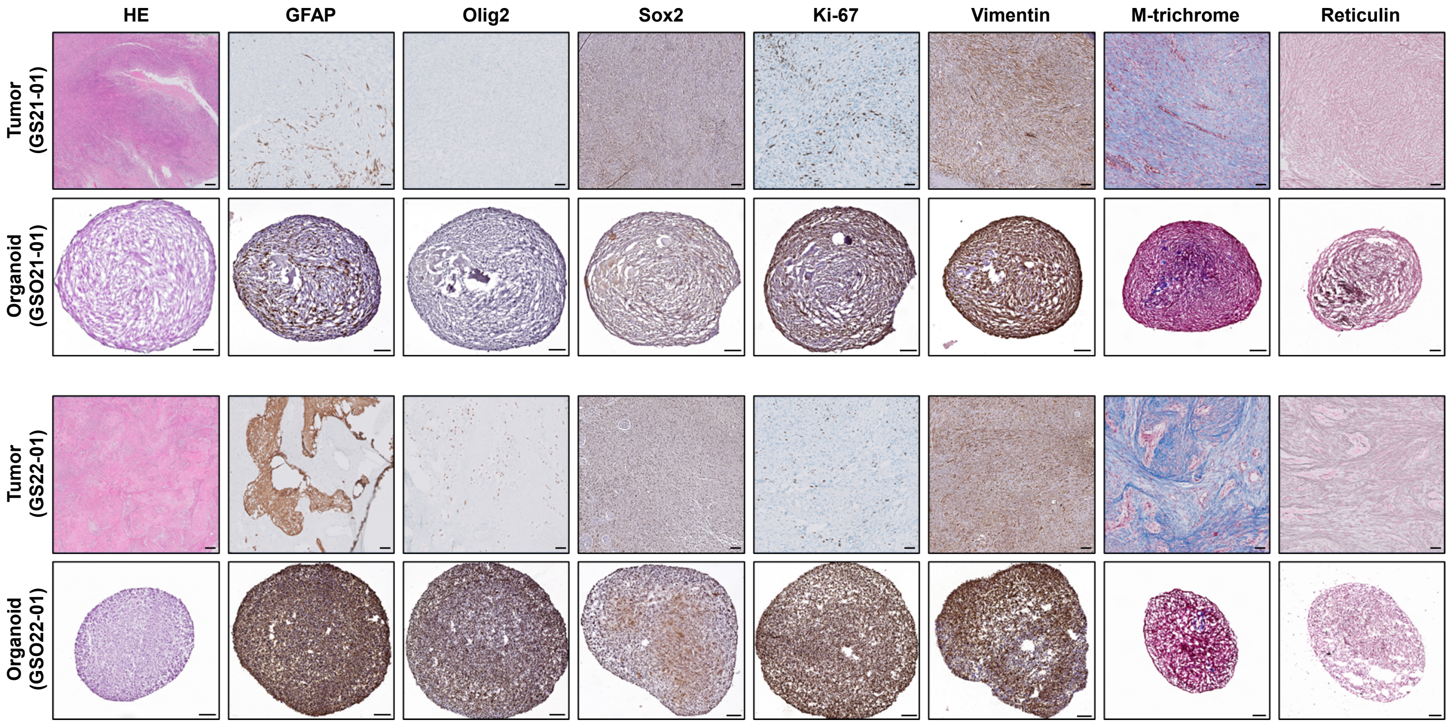

Fig. S1. Histological images of GSOs and their parental tumors (lower magnification of Fig. 1). Representative H&E and IHC images of GS tissues and GSOs at low magnificiation (×8). All images include a scale bar representing 100 µm. Staining was performed using GFAP, Olig2, and Sox2 to identify glial lineage; Ki-67 to evaluate proliferative activity; vimentin and reticulin to highlight mesenchymal and extracellular matrix components; and Masson's trichrome to visualize collagen deposition.

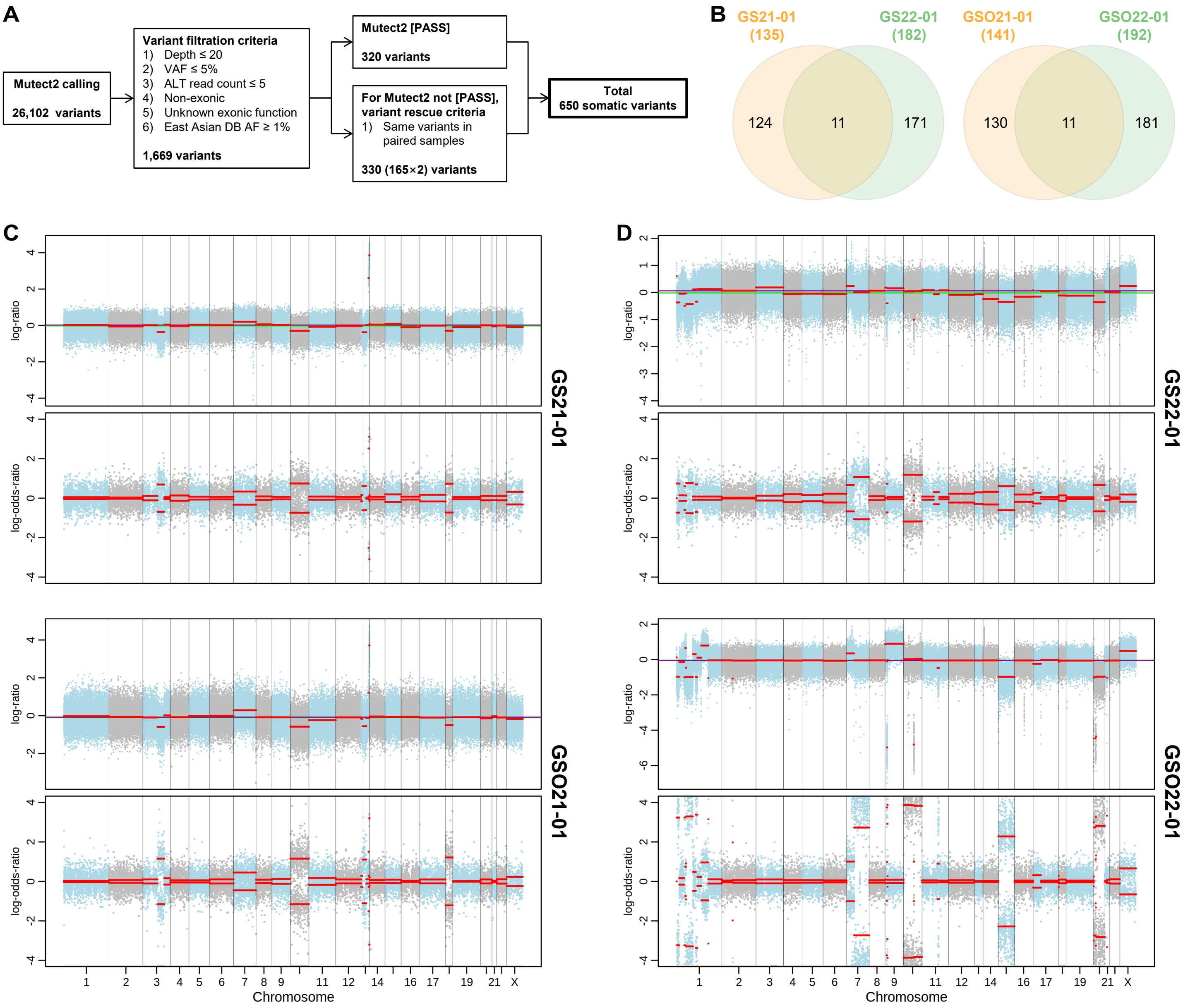

Fig. S2. WES of GSOs and corresponding parental tumors. (**A**) Summary of variant calling and refinement procedure. (**B**) Venn diagrams showing the number of shared and unique variants between parental tissues (left) and GSOs (right). (**C**, **D**) CNA profiles across the genome for GS21-01 and GSO21-01 (**C**) and GS22-01 and GSO22-01 (**D**), generated using FACETS.

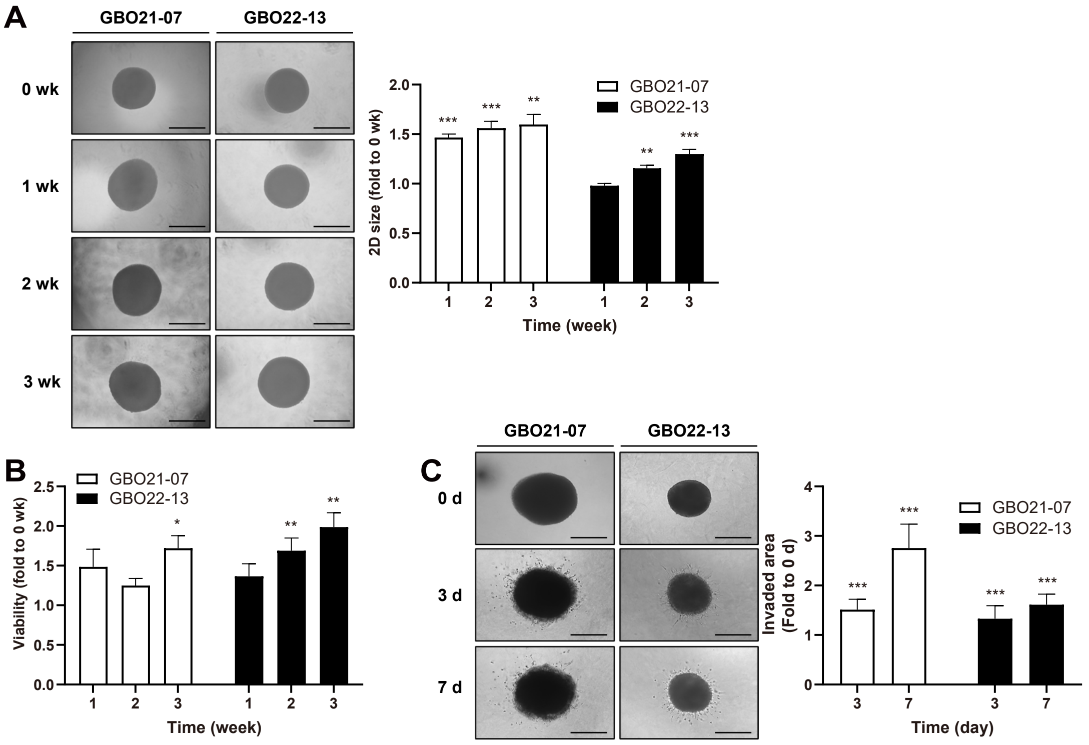

Fig. S3. Functional characteristics of GBOs. (**A**) Representative bright-field microscopy images of individual growing GBOs over a 3-weeks culture period. The two-dimensional size of the GBOs were quantified using ToupView software (n = 8, GBO21-07; n = 10, GBO22-13). (**B**) GBO viability was assessed weekly over 3 weeks using the WST assays (n = 5, GBO21-07; n = 10, GBO22-13). (**C**) Invasiveness of individual GBOs was measured at 3 d and 7 d using 3D invasion assays, and representative images were captured by bright-field microscopy. The invaded areas were quantified using ToupView software (n = 10, GBO21-07; n = 15, GBO22-13). For (**A**-**C**), all bright-field images include scale bars representing 500 μm, and repeated measured ANOVA was performed to evaluate statistical significance compared with 0 wk or 0 d control (*$P < 0.05$, **$P < 0.01$, ***$P < 0.001$).

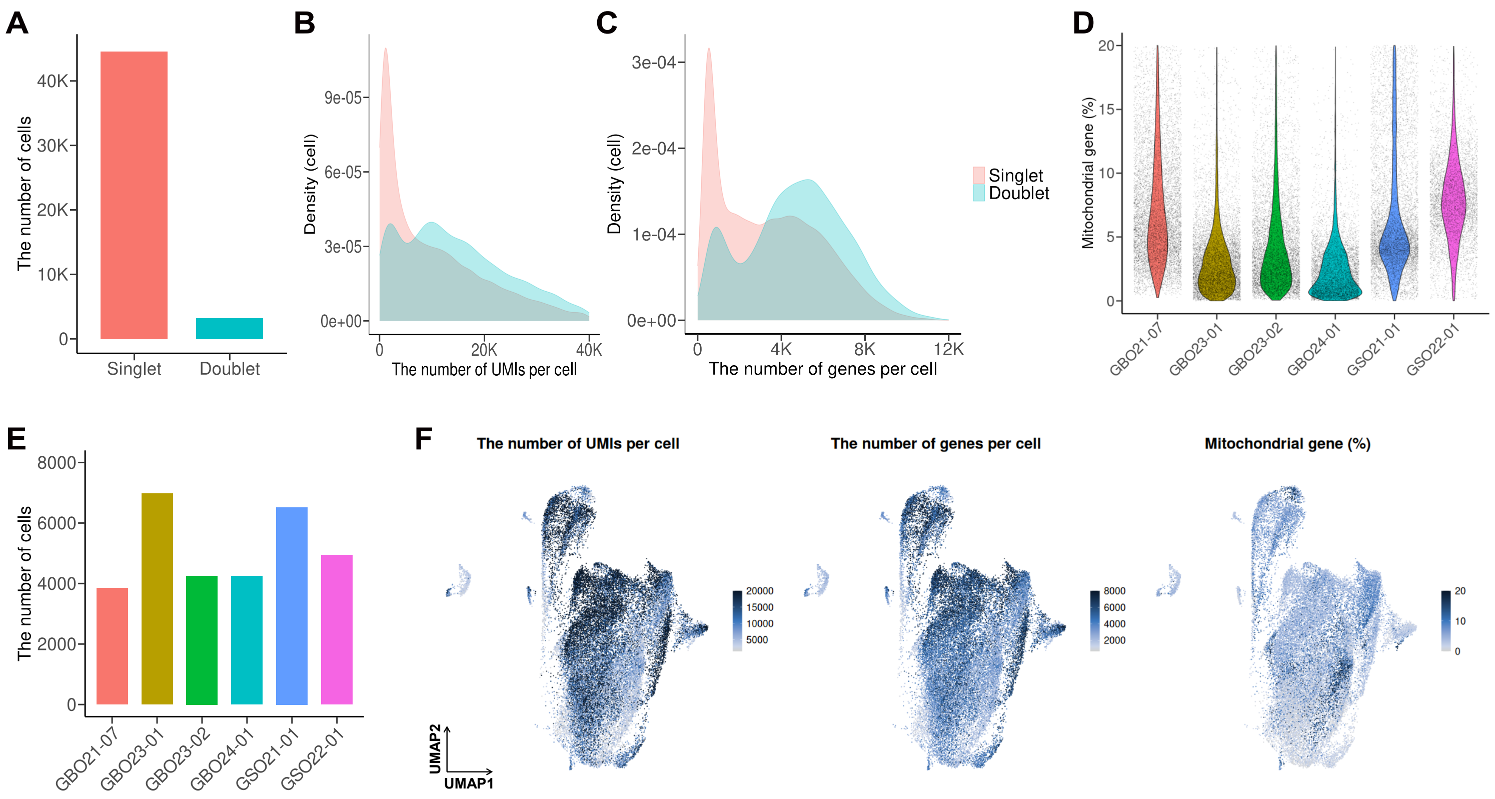

Fig. S4. Quality control of scRNA-seq data. (**A**) The number of singlet and doublet cells inferred by DoubletFinder. (**B**, **C**) Density plots of the number of UMIs (**B**) and the number of expressed genes (**C**) in the singlet and doublet cells. (**D**) Violin plots of the percentage of expressed mitochondrial genes in each sample. (**E**) The number of cells in each sample after the filtering for quality control. (**F**) UMAP plots colored by the number of UMIs (left), the number of expressed genes (center), and the percentage of mitochondrial genes (right).

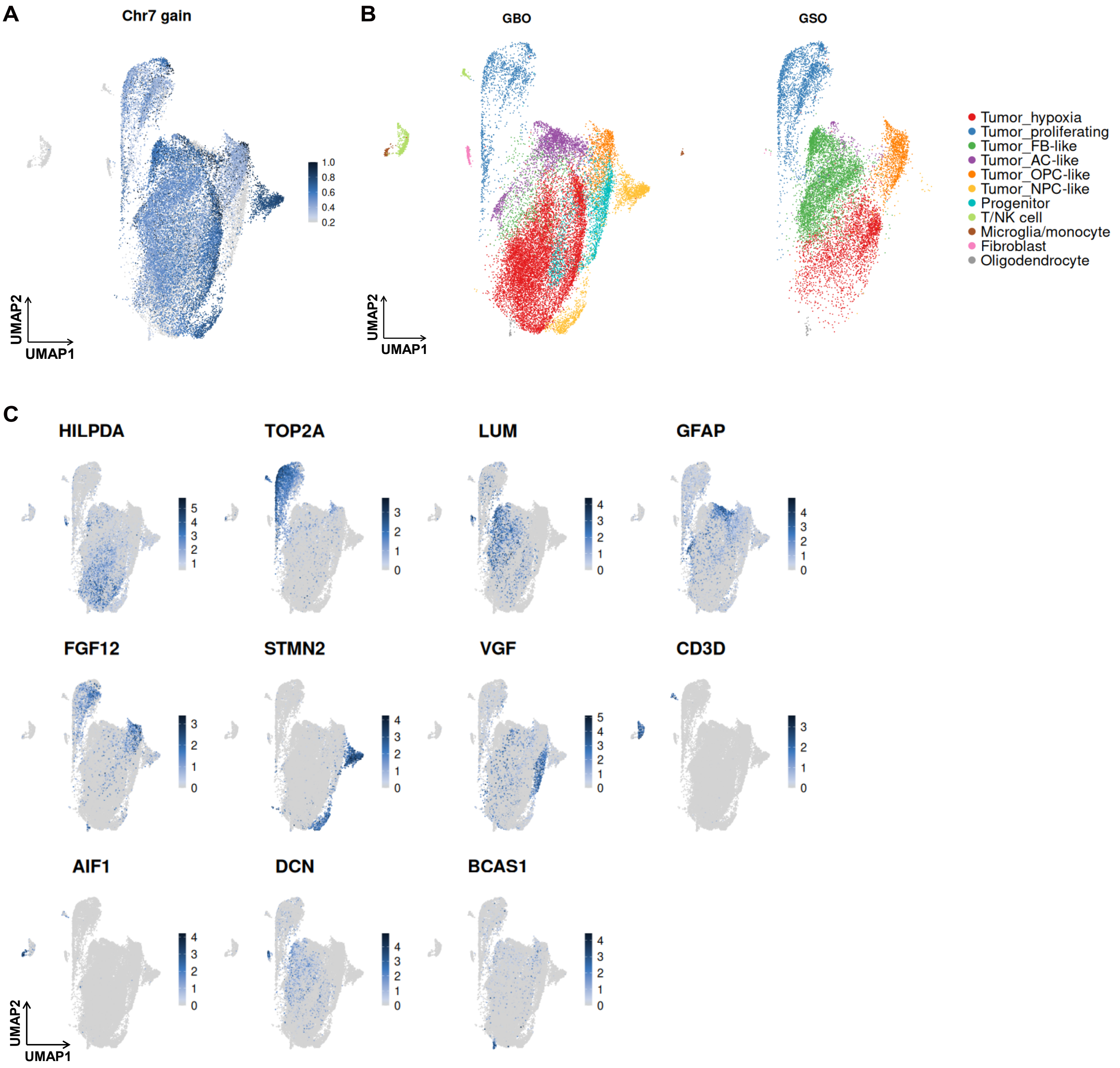

Fig. S5. UMAP plots for clustering and tumor cell identification in scRNA-seq data. (**A**) UMAP projection based on inferred CNA profiles (chromosome 7 gain). (**B**) UMAP plots showing cell clustering for each group separately. (**C**) UMAP plots showing the expression of representative marker genes for each cluster.

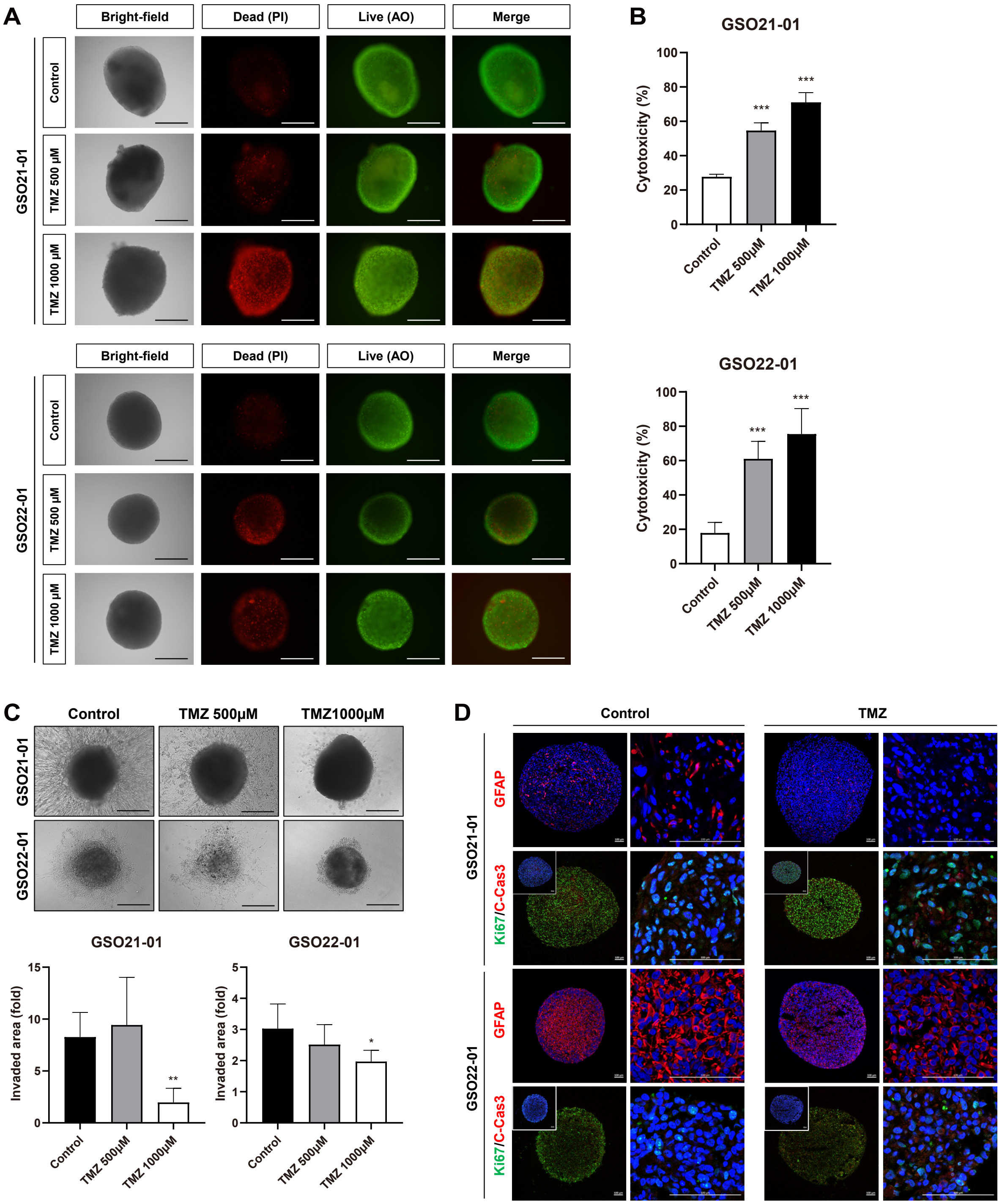

Fig. S6. TMZ-induced cytotoxic effects in GSOs. (**A**) Live/dead fluorescence staining of GSOs treated with TMZ. Dead cells were labeled with PI (red), while viable cells were labeled with AO (green). (**B**) LDH assays quantifying cytotoxicity in GSOs following TMZ treatment. One-way ANOVA was performed with Dunnett's *post hoc* test (n = 10, GSO21-01; n = 8, GSO22-01, ***$P < 0.001$). (**C**) 3D invasion assays using a collagen-Matrigel matrix to assess invasiveness of GSOs post-TMZ treatment. One-way ANOVA was performed with Dunnett's *post hoc* test (n = 6; *$P < 0.05$; **$P < 0.01$). (**D**) Representative IF images displaying GFAP, Ki-67, and cleaved caspase-3 expression in GSOs after TMZ exposure. Cell nuclei were counter-stained with Hoechst (blue). Left panels: lower magnification (×5); right panels: higher magnification (×40). Scale bar: 100 μm.

Table S1. The information of antibodies used for immunostaining.

Antibody	Dilution	Source	Catalog
Mouse monoclonal anti-Ki-67	IF: 1:100	Abcam	Cat# ab245113
Rabbit polyclonal anti-Ki-67	DAB: 1:2000<br>IF: 1:500	Abcam	Cat# ab15580
Rabbit monoclonal anti-Vimentin	DAB: 1:5000<br>IF: 1:800	Cell Signaling technology	Cat# 5741
Rabbit monoclonal anti-Cleaved caspase-3	IF: 1:500	Cell Signaling technology	Cat# 9664
Mouse monoclonal anti-GFAP	DAB: 1:2000<br>IF: 1:700	Cell Signaling technology	Cat# 3670
Rabbit monoclonal anti-Olig2	DAB: 1:100	Abcam	Cat# ab109186
Rabbit polyclonal anti-SOX2	DAB: 1:800	Abcam	Cat# ab97959
Mouse monoclonal anti-CD68	IF: 1:100	Abcam	Cat# ab955
Dako REAL EnVision Detection System, Perox/DAB+		Agilent Technologies	Cat# K500711-2
Goat anti-rabbit IgG-FITC	1:1000	Santa Cruz Biotechnology	Cat#sc-2012
Goat anti-mouse IgG-CFL 647	1:1000	Santa Cruz Biotechnology	Cat#sc-362287

Table S2. Overview of somatic mutations and key metrics from WES data.

Sample	Reads	Coverage mean	Coverage std	Total	Shared	Unique
GS21-01_N	62,125,791	115.9287	74.2104	-	-	-
GS21-01	67,460,870	122.7935	96.6469	135	124	11
GSO21-01	67,928,459	127.7616	109.7594	141	124	17
GS22-01_N	73,213,613	145.3145	92.7456	-	-	-
GS22-01	65,642,892	132.2781	87.8394	182	164	18
GSO22-01	68,588,866	139.1708	99.0623	192	164	28
